# Editorial: Molecular and cellular mechanisms of synaptopathies: emerging synaptic aging-related molecular pathways in neurological disorders

**DOI:** 10.3389/fnagi.2023.1267033

**Published:** 2023-08-10

**Authors:** Hector Ramiro Quintá, Laura Ibanez, Carlos Cruchaga, Dario Maschi

**Affiliations:** ^1^National Scientific and Technical Research Council (CONICET), Buenos Aires, Argentina; ^2^Laboratorio de Medicina Experimental “Dr. J. Toblli”, Hospital Aleman, Buenos Aires, Argentina; ^3^Rehabilitation Medicine Department, University of Minnesota Twin Cities, St. Paul, MN, United States; ^4^Department of Neurology, School of Medicine, Washington University in St. Louis, St. Louis, MO, United States; ^5^Department of Psychiatry, School of Medicine, Washington University in St. Louis, St. Louis, MO, United States; ^6^NeuroGenomics and Informatics Center, Washington University School of Medicine, St. Louis, MO, United States; ^7^Hope Center for Neurological Disorders, School of Medicine, Washington University in St. Louis, St. Louis, MO, United States; ^8^Department of Cell Biology and Physiology, School of Medicine, Washington University in St. Louis, St. Louis, MO, United States

**Keywords:** neurodegenerative diseases, synaptopathies, synaptic aging, Alzheimer's disease, biomarkers, therapeutic targets

Despite the enormous advances made in our understanding of neurological disorders, a knowledge gap still exists that prevents the scientific community from linking clinical manifestations with actual cellular neuropathological changes. This connection is especially crucial for pathologies like Alzheimer's disease (AD), which has a 10–20 year preclinical period offering an extended window for potential interventions (McKhann et al., 1984; Sperling et al., 2011). However, the preclinical period has been challenging to explore due to the lack of specific prognostic and predictive biomarkers.

To identify disease progression biomarkers, we must diligently explore and identify new causal genes and pathways associated with the disease. Furthermore, by understanding these pathologies' mechanistic, we will speed up the development of new disease-modifying therapies. Future advances in this endeavor will necessitate the collaborative efforts of researchers from diverse backgrounds. This diversity is reflected in our editorial panel and among the contributors to this topic. Our Frontiers Research Topic, entitled “*Molecular and cellular mechanisms of synaptopathies: emerging synaptic aging-related molecular pathways in neurological disorders*”, comprises original research articles from experts in the neuroscience field. These experts explore novel brain disease-related pathways that could serve as potential therapeutic targets. The number and originality of these contributions highlight the interest and ongoing activity in this area of investigation.

Among the promising new pharmacological discoveries, Bellanti et al. highlights the therapeutic potential of ultramicronized palmitoylethanolamide (um-PEA) in AD. Chronic um-PEA treatment improved mitochondrial function and restored energy metabolism in the frontal cortex of mice, suggesting um-PEA treatment as a potential novel strategy for future clinical treatment of AD due to its bioenergetic effects.

Nan et al. offer valuable insights regarding the intersection of metabolic disorders and neurodegenerative diseases. Increasing evidence points toward a potential connection between diabetes and AD. Diabetes can lead to cognitive impairment and brain changes such as altered synaptic plasticity, aggregated amyloid-beta (Aβ) plaques, and neurofibrillary tangles composed of hyperphosphorylated tau protein. Given the demographic trend toward global aging and that the incidence of diabetes has been steadily rising, the significance of their research is clear. They examine the pathogenesis of post-menopausal diabetic encephalopathy and propose Forsythoside·B as an effective a therapeutic agent.

Expanding our understanding of the role of age in cognitive health, Li et al. present a novel research perspective on the impact of young plasma on anesthesia and surgery-induced cognitive impairment in aged rats. Their study reveals a compelling connection between young plasma preinfusion and a reduction in such cognitive impairments. Thus, young plasma emerges as a potential therapeutic strategy to mitigate cognitive dysfunction linked to surgery and anesthesia in the aged population.

A remarkable example of a promising study that uncovers new treatment possibilities for neurodegenerative pathologies is the work of León et al.. They explore the potential of c-Abl tyrosine kinase as a therapeutic target in AD pathogenesis. In previous work, Dr. Alvarez, the lead author, had collaborated with Dr. Marugan from the National Center for Advancing Translational Sciences (NCATS) to develop neurotinib, a novel c-Abl inhibitor capable of crossing the blood-brain barrier. Here, they demonstrate that down-regulating c-Abl leads to improved cognitive performance and reduced neuropathological symptoms in AD mouse models, marking neurotinib as a promising candidate for AD therapy.

Delving into the intricate mechanisms behind protein misfolding, Cazzaro et al. contribute significantly to our understanding of the disease propagation in AD. They study the mechanisms that govern the secretion of small extracellular vesicles containing misfolded proteins. Their findings reveal that Slingshot Homolog-1 augments this secretion, suggesting a promising approach to promoting the degradation of misfolded proteins and curbing the spread of intercellular pathology.

In an effort to understand how AD pathology affects neuronal activity, Martinsson et al. have shown that elevated levels of Aβ and its precursor protein, the amyloid precursor protein (APP), induce increased neuronal activity in AD. Specifically, they found impaired adaptation of calcium transients to global activity changes and observed that neurons failed to adjust the length of their axon initial segments, which typically affects excitability. They hypothesize that the close localization of APP and Aβ near synapses may play a vital role in the altered synaptic responses. These insights point to potential treatment strategies focusing on early Aβ/APP-induced hyperexcitability and synapse dysfunction.

Underscoring the importance of synaptic health in neurodegenerative disease, Olajide et al. show that amyloid beta peptide 1–42 causes significant mitochondrial dysfunction at glutamatergic synapses, leading to rapid synapse alterations, reduced energy production efficiency, and a significant reduction in key mitochondrial and synaptic protein expression. Notably, they show that lowering reactive oxygen species prevents synaptic impairments. This implies that therapies targeting reactive oxygen species might help curb the advancement of neurodegeneration in chronic models of AD.

Zhang et al. shift our attention to the protective potential of naturally occurring compounds in neurodegenerative diseases. They show that polyphenols in oolong tea have neuroprotective and anti-aging activities, nominating them as potential therapeutic agents for age-related neurodegenerative diseases.

Pursuing further insights into the role of protein aggregates, Ferrari et al. shed light on the impact of soluble α-synuclein oligomers in Parkinson's disease, finding that these oligomers play a central role in the early events leading to synaptic loss. This discovery could offer new avenues for early detection and potential therapeutic interventions, underscoring the importance of comprehending the role of these oligomers in the onset of Parkinson's disease and related disorders.

As we conclude this brief overview of the featured articles in our Research Topics, we want to draw attention to the work by Carbonell et al.. They used quantitative mass spectrometry to compare hippocampal synaptic proteomes across different Autism Spectrum Disorder (ASD) mouse models, identifying shared alterations in cellular and molecular pathways at the synapse. These results suggest that diverse ASD-related genes may converge on shared synaptic signaling pathways, paving the way for a better understanding of the pathogenesis of not just ASD but also other neuropathologies.

Addressing neurodegenerative diseases remains a critical challenge of our era, further intensified by an aging population, increasing disease prevalence, and lack of disease-modifying therapies and good biomarkers. Vital to mitigating these conditions is strategically targeting the early stages of disease progression to prevent irreversible neuronal loss and increase therapeutic effectiveness. However, this is often hindered by the fact that their symptoms frequently remain undetected until the later stages, and our knowledge of the molecular events that initiate these diseases is still limited. Despite these difficulties, the innovative approaches and unwavering commitment demonstrated by the researchers contributing to this topic reflect the ongoing strides made to unravel these diseases' complexities and propose out-of-the-box therapeutic targets advancing the field toward personalized medicine. As we continue to gain novel insights and explore new pathways, we are confident in our collective ability to advance our understanding and ultimately turn the tide against neurodegenerative diseases.

## Author contributions

HRQ: Writing—original draft, Writing—review and editing. LI: Writing—original draft, Writing—review and editing. CC: Writing—original draft, Writing—review and editing. DM: Writing—original draft, Writing—review and editing.
